# Arginine-rich cell-penetrating peptide-modified extracellular vesicles for active macropinocytosis induction and efficient intracellular delivery

**DOI:** 10.1038/s41598-017-02014-6

**Published:** 2017-05-16

**Authors:** Ikuhiko Nakase, Kosuke Noguchi, Ayako Aoki, Tomoka Takatani-Nakase, Ikuo Fujii, Shiroh Futaki

**Affiliations:** 1 Nanoscience and Nanotechnology Research Center, Research Organization for the 21st Century, Osaka Prefecture University, 1-2, Gakuen-cho, Naka-ku, Sakai, Osaka, 599-8570 Japan; 2 Graduate School of Science, Osaka Prefecture University, 1-1, Gakuen-cho, Naka-ku, Sakai, Osaka, 599-8531 Japan; 3 Department of Pharmaceutics, School of Pharmacy and Pharmaceutical Sciences, Mukogawa Women’s University, 11-68, Koshien Kyuban-cho, Nishinomiya, Hyogo, 663-8179 Japan; 4 Institute for Chemical Research, Kyoto University, Uji, Kyoto, 611-0011 Japan

## Abstract

Extracellular vesicles (EVs) including exosomes have been shown to play crucial roles in cell-to-cell communication because of their ability to carry biofunctional molecules (e.g., microRNAs and enzymes). EVs also have pharmaceutical advantages and are highly anticipated to be a next-generation intracellular delivery tool. Here, we demonstrate an experimental technique that uses arginine-rich cell-penetrating peptide (CPP)-modified EVs to induce active macropinocytosis for effective cellular EV uptake. Modification of arginine-rich CPPs on the EV membrane resulted in the activation of the macropinocytosis pathway, and the number of arginine residues in the peptide sequences affected the cellular EV uptake efficiency. Consequently, the ribosome-inactivating protein saporin-encapsulated EVs modified with hexadeca-arginine (R16) peptide effectively attained anti-cancer activity.

## Introduction

Extracellular vesicles (EVs) including exosomes (30–200 nm in diameter) are cell-secreted vesicles with a lipid bilayer. Most cells constitutively secrete EVs, which are abundant in bodily fluids, including blood, saliva, urine, and breast milk^1–3^. EVs carry genetic materials (e.g., microRNAs) and enzymes to other cells, which leads to cell regulation via the EV contents and modulation of the immune response in cell-to-cell communication^1–5^. EVs are also highly anticipated as the next-generation therapeutic carriers because of their pharmaceutical advantages, including the 1) effective usage of cell-to-cell communication routes, 2) absence of cytotoxicity, 3) controlled immunogenicity, 4) constitutive secretion, 5) encapsulation of additional biofunctional molecules, and 6) expression of functional proteins in membranes^6^. However, a well-developed methodology for increasing the cellular uptake efficiency of EVs is necessary to achieve effective intracellular delivery of EV contents, especially in the cytosol. A considerable number of EVs are secreted into bodily fluids (approximately 3,000,000 exosomes/μl in the blood)^1–3^, which results in cellular EV uptake competition. The negative charge of the EV membrane also prevents them from accumulating on negatively charged cellular membranes^7, 8^.

However, our research group recently reported that the active induction of macropinocytosis (accompanied by actin reorganization, ruffling of plasma membrane, and engulfment of large volumes of extracellular fluid)^9, 10^ by cancer-related receptors (e.g., epidermal growth factor receptor) and the expression of oncogenic K-Ras significantly enhance the cellular uptake efficiency of EVs^7^. Therefore, macropinocytosis induction by the functionalized EV itself is strongly considered to be useful for the EV-based intracellular delivery of therapeutic molecules.

**Figure 1:**
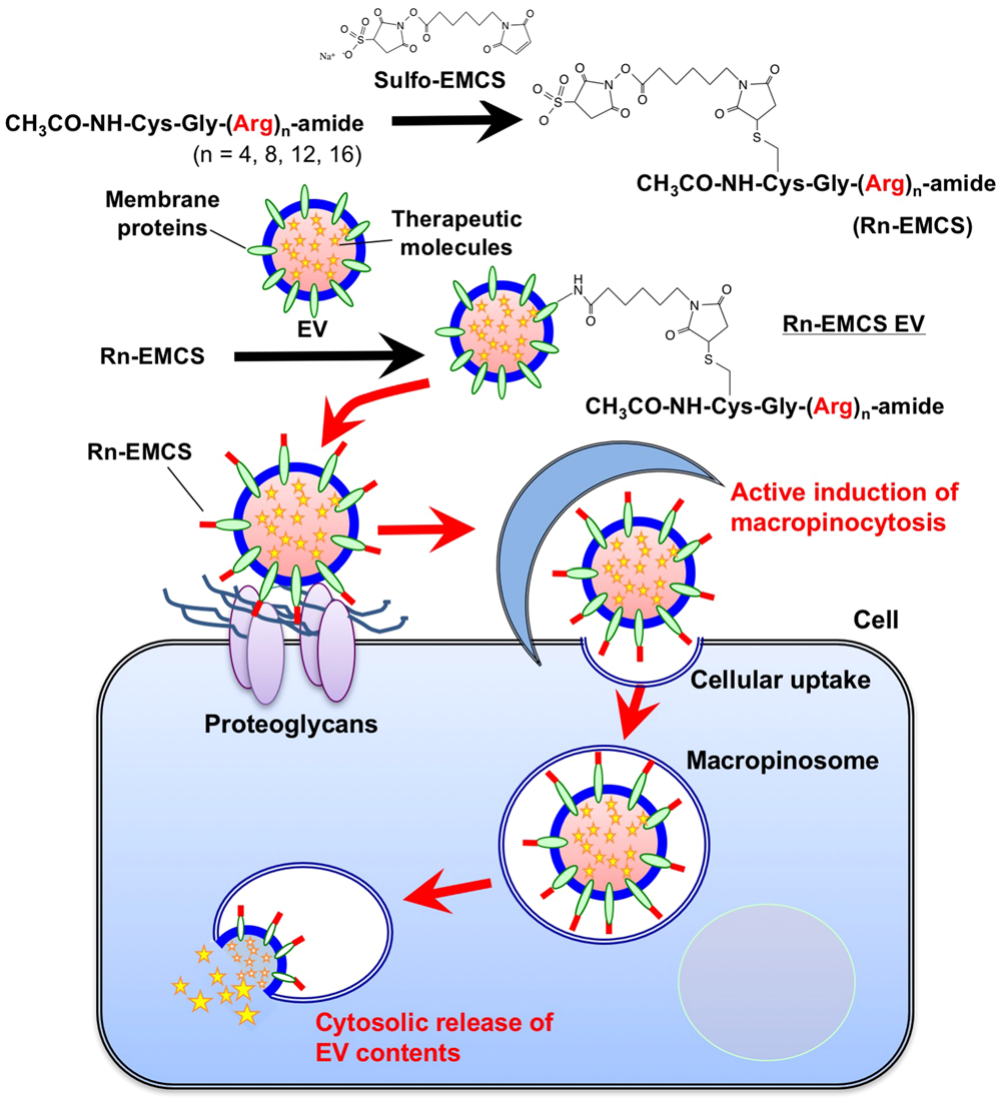
Schematic representation of the cellular uptake of EVs modified by oligoarginine peptides. Objective EVs were conjugated with oligoarginine peptides via a sulfo-EMCS linker. Oligoarginine peptide-modified EVs actively induce macropinocytosis, thereby leading to their efficient cellular uptake.

Recently, we demonstrated that the modification of EVs with octaarginine peptide, which is a representative arginine-rich cell-penetrating peptide (CPP), results in the effective induction of macropinocytosis and uptake of cellular EVs^11^. Arginine-rich CPPs, including human immunodeficiency virus type 1 (HIV-1) TAT (48–60) peptide and oligoarginine peptides, have been shown to be efficiently internalized by cells, and the CPPs have been reported to be promising carriers for the intracellular delivery of various bioactive molecules, such as proteins, peptides, and nucleic acids^12, 13^. Macropinocytosis has also been shown to be an important pathway for the physiological cellular uptake of arginine-rich CPPs^14–18^. Octaarginine peptide, which is a representative arginine-rich CPP, has been shown to induce clustering of syndecan-4 proteoglycan on plasma membranes, which results in the binding of PKCα to the V domain of the proteoglycan in the cytosol^19^. The induction of proteoglycan clustering and PKCα binding results in macropinocytosis induction and cellular uptake of the peptide^19^. As previously mentioned, the modification of EV membranes with octaarginine peptides results in increased cellular EV uptake^11^. However, the number of arginine residues in the sequence of oligoarginine peptides has been shown to influence their cellular uptake and cytosolic release efficiency^20^. Therefore, in this research, we studied how modifying the EV membranes using oligoarginine peptides with a different number of arginine residues in the peptide sequence impacts macropinocytosis induction, cellular EV uptake, and cytosolic release of EV contents. EV membranes were modified with oligoarginine peptides that each had different numbers of arginine residues (Rn: n = 4, 8, 12, 16), which was achieved by mixing with Rn-EMCS (N-ε-malemidocaproyl-oxysuccinimide ester), an amine-to-sulfhydryl crosslinker (Fig. 1, Supplementary Table 1).

## Results

### Preparation of Rn-EMCS-modified EVs and cytotoxicity assessment

CD63 is a marker membrane tetraspanin protein of the EV (exosome), and in this study, HeLa cells stably expressing green fluorescent protein (GFP)-fused CD63 (CD63-GFP-HeLa) (Supplementary Fig. 1a) were prepared to secrete CD63-GFP-expressing EVs (CD63-GFP- EVs). The secreted CD63-GFP EVs were collected and isolated from the cell culture medium via ultracentrifugation methods^21^. Vesicular structures of the isolated EVs were observed using transmission electron microscopy (TEM) (Supplementary Fig. 1b). Moreover, the expression levels of the EV (exosome) marker proteins CD9 and CD63 were detected using western blot analysis (Supplementary Fig. 1c).

Oligoarginine peptides were modified on EV membranes by mixing with Rn-EMCS (Fig. 1), as described in the Methods section. We have already reported that oligoarginine peptides equipped with a sulfosuccinimidylsuberyl moiety allow easy modification of targeted cargo molecules with peptides via amino moiety^22^. Before conducting the cellular EV uptake assay, we tested the cytotoxicity of Rn-EMCS-modified EVs (20 μg/ml, 1.1 × 10^8^ EV particles/ml) on CHO-K1 cells (derived from Chinese hamster ovaries) for 24 h at 37 °C in 10% fetal bovine serum (FBS)-containing medium prior to the WST-1 (4-[3-(4-iodophenyl)-2-(4-nitrophenyl)-2H-5-tetrazolio]-1,3-benzene disulfonate) analysis and microscope observation (Supplementary Fig. 2). Almost no cytotoxicity was observed with the treatment of each Rn (n = 4, 8, 12)-EMCS (5–20 μM)-conjugated EV (20 μg/ml); however, high toxicity was observed with the R16-EMCS (20 μM)-conjugated EV (20 μg/ml) (cell viability: 70%) analysed using WST-1 assay (Supplementary Fig. 2). In the case of the R16-EMCS (10 μM)-conjugated EV (20 μg/ml), cell viability was not affected (Supplementary Fig. 2). Therefore, this peptide concentration was set in the experimental condition of R16-EMCS (10 μM) conjugation.

### Effects of modification of Rn-EMCS on EV membrane on cellular uptake

**Figure 2:**
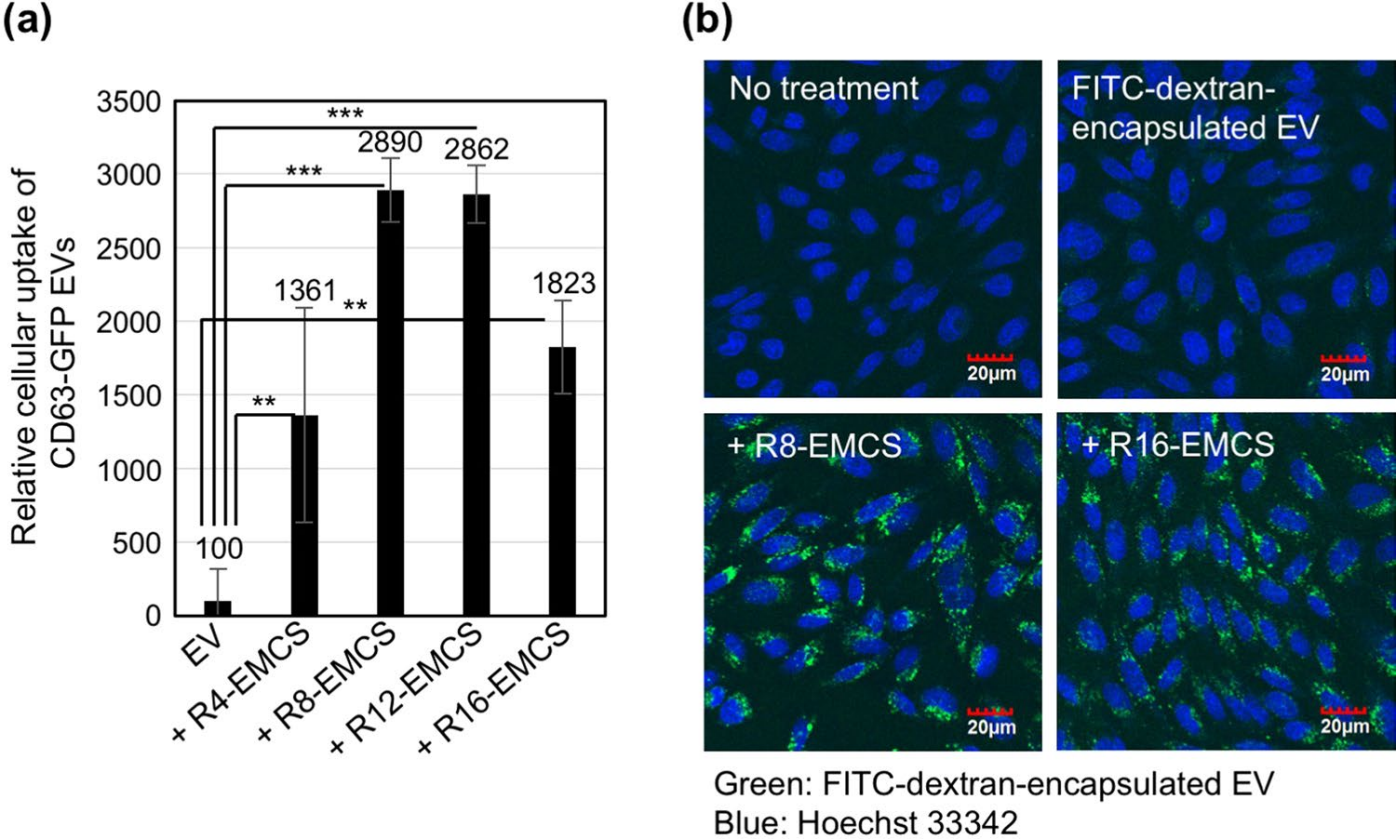
Enhanced cellular uptake efficiency of EVs upon modification with oligoarginine peptides. (**a**) Relative cellular uptake of CD63-GFP EVs (20 μg/ml) modified with Rn-EMCS (n = 4, 8, 12: 20 μM, n = 16: 10 μM) in CHO-K1 cells for 24 h at 37 °C according to a flow cytometry analysis. The data are expressed as the average (±SD) of three experiments. **p < 0.01, ***p < 0.001. (**b**) Confocal microscopy observations of CHO-K1 cells treated with FITC-dextran-encapsulated EVs (20 μg/ml) modified with Rn-EMCS (n = 8: 20 μM, n = 16: 10 μM) under the same experimental conditions as (**a**) (green: FITC-dextran-encapsulated EVs, blue: Hoechst 33342). Scale bar: 20 μm.

Figure 2a shows the relative cellular uptake of Rn-EMCS-modified CD63-GFP-EVs (20 μg/ml) in 10% serum-containing cell culture medium for 24 h at 37 °C into the CHO-K1 cells based on a flow cytometry analysis. Conjugation of oligoarginine peptides on EV membranes significantly enhanced the cellular EV uptake (Fig. 2a). The number of arginine residues in the conjugated oligoarginine peptides affected the cellular uptake efficiency. The conjugation of R8-EMCS or R12-EMCS resulted in the highest cellular uptake of all the oligoarginine-peptide conjugations, and the cellular EV uptake was 29-fold higher because of the modification of R8- or R12-EMCS (Fig. 2a). When the cells were treated with CD63-GFP-EVs premixed with R8 peptide without the EMCS linker, the cellular EV uptake efficiency did not increase (Supplementary Fig. 3), suggesting that the EMCS linker is essential for the modification of oligoarginine peptides on exosomal membranes. We also examined the cellular uptake of cargo molecules artificially encapsulated in EVs (Fig. 2b, Supplementary Fig. 4). We prepared fluorescein-5-isothiocyanete (FITC)-dextran (molecular weight: 70,000)-encapsulated EVs via electroporation^7, 8, 11^. Figure 2b shows confocal microscopy observations of the CHO-K1 cells treated with FITC-dextran-encapsulated EVs for 24 h at 37 °C. Modifying the EV membranes with oligoarginine peptides enhanced their cellular uptake (Fig. 2b). The fluorescent intensity in the cells under the same experimental conditions was analysed using a flow cytometer (Supplementary Fig. 4). In addition, the increased fluorescent intensity from the FITC-dextran-encapsulated EVs conjugated with R8-EMCS or R16-EMCS indicated the efficient uptake by cells (Fig. 2b, Supplementary Fig. 4). Supplementary Fig. 5 also shows the comparison of cellular uptake efficacy on EVs decorated with R8-EMCS (20 μM) or R16-EMCS (10 μM) at different protein concentrations of EVs (1~20 μg/ml), and their cellular uptake efficacy increased in a concentration-dependent manner of EVs.

Fluorescent-labelled peptides (FITC-Rn-EMCS) were also used to assess the binding of the oligoarginine peptides to the EV membrane using a spectrofluorometer, and this method resulted in the binding of FITC-R4-EMCS (9.9 μM), FITC-R8-EMCS (4.1 μM), FITC-R12-EMCS (1.5 μM) or FITC-R16-EMCS (1.0 μM) to 20 μg/ml EVs. In the case of FITC-R8 or FITC-R16 without an EMCS linker, this method resulted in the binding of FITC-R8 (8.6 nM) or FITC-R16 (26.1 nM) to 20 μg/ml EVs. The zeta-potential of the R8-EMCS- or R16-EMCS-modified EV was 10.8 mV (modification of R8-EMCS) or −0.2 mV (modification of R16-EMCS), and the average diameter of the EVs was 87.2 ± 19.3 nm (modification of R8-EMCS) or 104.8 ± 25.2 nm (modification of R16-EMCS), as determined using the zeta potential and a particle size analyser. The average diameter of the original isolated EVs that were not modified by Rn-EMCS was 102.3 ± 17.3 nm, and the zeta potential of isolated EVs was −6.4 mV, which suggested an increase in the EV membrane charge via the modification of oligoarginine peptides. TEM observations of the R8-EMCS- or R16-EMSC-modified EVs showed vesicular structures similar to those of the originally isolated EVs that were not modified by oligoarginine peptides (Supplementary Fig. 6).

### Active induction of macropinocytosis by Rn-EMCS-modified EVs

**Figure 3:**
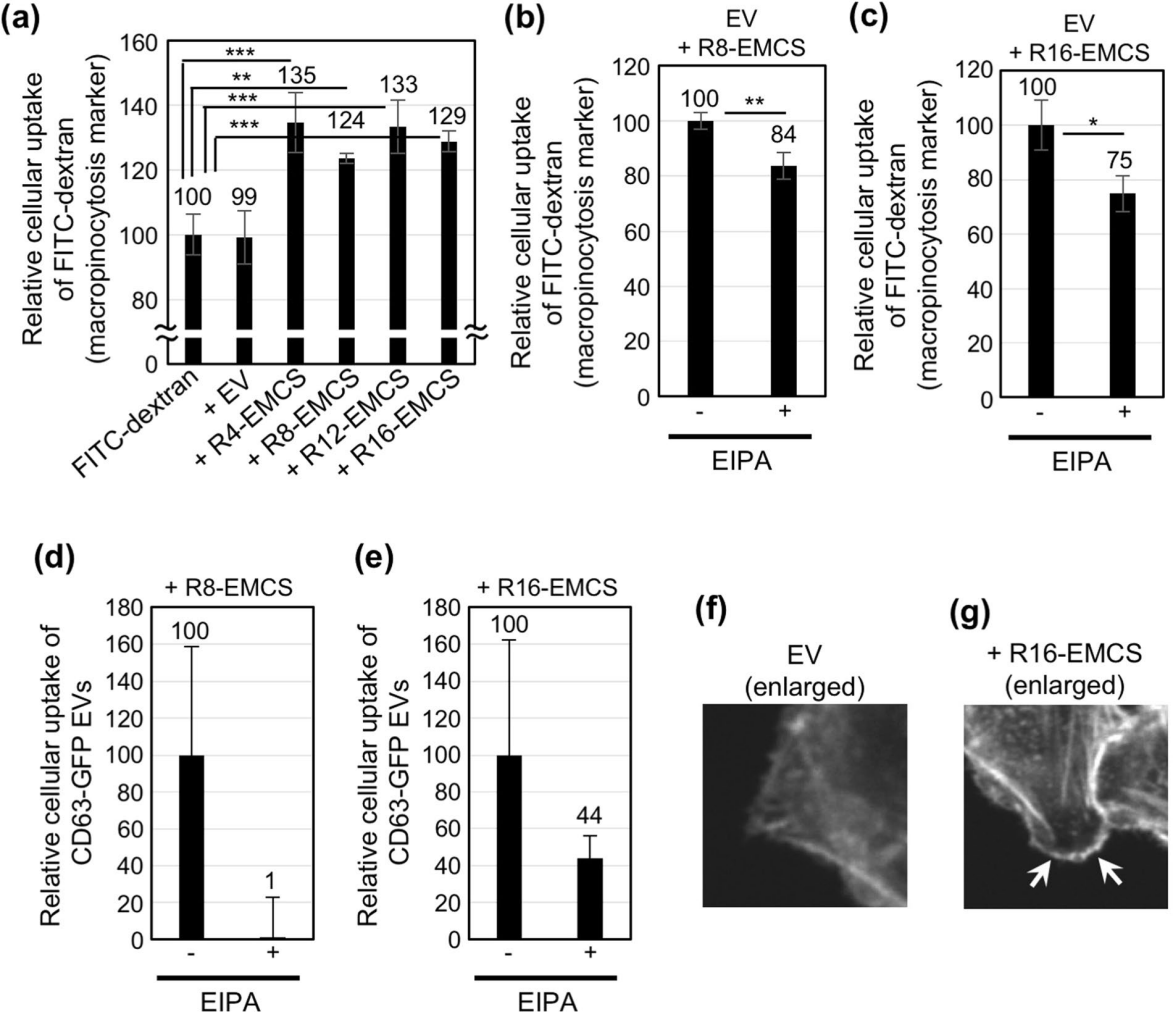
Active induction of macropinocytosis by the modification of EV membranes with oligoarginine peptides. (**a**) Relative cellular uptake of the macropinocytosis marker FITC-dextran in the presence or absence of EVs (20 μg/ml) with or without modification by Rn-EMCS (n = 4, 8, 12: 20 μM, n = 16: 10 μM) for 3 h at 37 °C according to a flow cytometry analysis. (**b**,**c**) Relative cellular uptake of the macropinocytosis marker FITC-dextran in the presence or absence of EVs (20 μg/ml) with modification by Rn-EMCS (n = 8: 20 μM (**b**), n = 16: 10 μM (**c**)) for 1 h at 37 °C with or without the treatment with the macropinocytosis inhibitor EIPA according to a flow cytometry analysis. The data are expressed as the average (±SD) of three experiments. *p < 0.05, **p < 0.01, ***p < 0.001. (**d**,**e**) Relative cellular uptake of CD63-GFP EVs (20 μg/ml) with modification by Rn-EMCS (*n* = 8: 20 μM (**d**), *n* = 16: 10 μM (**e**)) was conducted for 1 h at 37 °C with or without treatment with the macropinocytosis inhibitor EIPA according to a flow cytometry analysis. The data are expressed as the average (±SD) of three experiments. (**f**,**g**) Confocal microscope observation of CHO-K1 cells treated with EVs (20 μg/ml) modified with (**g**) or without (**f**) R16-EMCS (10 μM) for 20 min at 37 °C (enlarged pictures of Supplementary Fig. 8). Cellular staining with rhodamine-phalloidin was conducted to visualize F-actin prior to the observations.

To study the cellular uptake mechanisms of the oligoarginine peptide-modified EV, we tested the induction of macropinocytosis by the treatment with EVs on cells. Under low temperature (4 °C), which is an experimental condition for the prevention of endocytosis^20^, we tested the cellular EV uptake. Supplementary Fig. 7 shows the flow cytometry analysis of CHO-K1 cells treated with the R8-EMCS- or R16-EMCS-modified CD63-GFP EV for 2 h at 37 °C or 4 °C. The cellular uptake efficiency of the R8-EMCS- or R16-EMCS-modified CD63-GFP EV was greatly reduced in the low-temperature treatment compared with that in the 37 °C treatment (Supplementary Fig. 7), which indicates that energy-dependent endocytosis is important for the cellular uptake of the oligoarginine-modified EV. Next, we examined the active induction of macropinocytosis by the treatment with the oligoarginine peptide-modified EV analysed using FITC-dextran (molecular weight: 70,000), which is a marker of macropinocytotic cellular uptake^14, 23^. Figure 3a shows the results of the flow cytometry analysis of CHO-K1 cells treated with FITC-dextran in the presence or absence of oligoarginine peptide-modified EVs. The treatments with each of the oligoarginine peptide-modified EVs resulted in enhanced cellular uptake of the macropinocytosis marker of FITC-dextran (Fig. 3a). Conversely, the EVs that were not modified by oligoarginine peptides did not increase the cellular uptake of FITC-dextran (Fig. 3a), suggesting that the modification of the EV membranes with oligoarginine peptides enhanced the cellular uptake route of macropinocytosis. The cellular uptake of the macropinocytosis marker was decreased by treatment with the macropinocytosis inhibitor, EIPA^20^ (Fig. 3b and c). Figure 3d and e show the effects of EIPA on the cellular uptake of R8-EMCS or R16-EMCS-modified EVs; the EIPA treatment reduced cellular EV uptake, suggesting that macropinocytosis induction is important for enhanced cellular uptake of EVs modified with oligoarginine peptides. We also found that lamellipodia formation and membrane ruffling by actin organization occurred when the cells were treated with oligoarginine peptide-modified EVs (Fig. 3g, Supplementary Fig. 8). In the case of EVs without the peptide modification, active lamellipodia formation was not be observed (Fig. 3f, Supplementary Fig. 8), which suggested that the modification of EV membranes with oligoarginine peptides is important for the induction of membrane ruffling and macropinocytosis. As previously reported, proteoglycans expressing on plasma membranes play a crucial role for the effective cellular uptake of oligoarginine peptides^17, 19^. We further used the CHO-A745 cell line, which lacks xylosyltransferase, an enzyme necessary for the initiation of the glycosaminoglycan (GAG) synthesis, and does not produce detectable levels of any proteoglycans^17^. We found that the cellular EV uptake efficacy of both the R8-EMCS-modified and R16-EMCS-modified EVs was reduced in CHO-A745 cells in comparison to CHO-K1 cells (Supplementary Fig. 9), suggesting that proteoglycans play a crucial role in the cellular uptake of oligoarginine peptide-modified EVs.

### Enhanced biological activity of saporin encapsulated in EVs modified with Rn-EMCS

**Figure 4:**
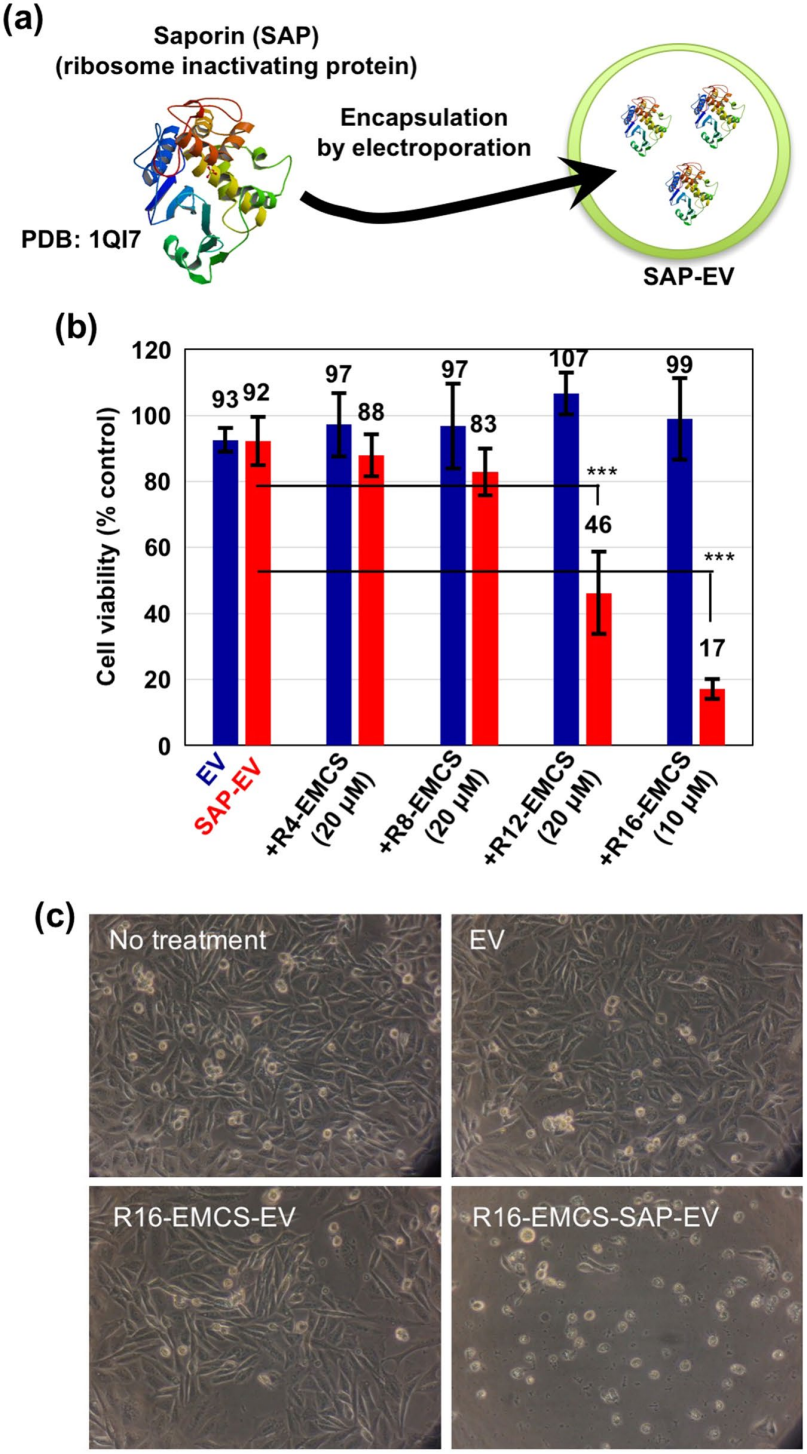
Increased anti-cancer activity of saporin encapsulated in EVs modified by Rn-EMCS. (**a**) Schematic representation of the encapsulation of saporin (SAP) (PDB (Protein Data Bank) accession number: 1QI7)^25^ in EVs by electroporation. (**b**) CHO-K1 cells were treated with SAP encapsulated in EVs (20 μg/ml) (EVs (25 μg) and SAP (50 μg) in electroporation condition as described in Methods section) with or without modification by Rn-EMCS (n = 4, 8, 12: 20 μM, n = 16: 10 μM) for 48 h at 37 °C. Cell viability was then analysed using a WST-1 assay. The data are expressed as the average (±SD) of four experiments. ***p < 0.001. (**c**) Microscope observations of CHO-K1 cells treated with EV samples under the same experimental conditions as (**b**).

To study the delivery of bioactive proteins based on EVs and cytosolic release, we encapsulated the ribosome-inactivating protein saporin^24, 25^, which functions as an anti-cancer drug, in EVs via electroporation, and this experimental method is similar to that used to encapsulate the FITC-dextran in EVs (Fig. 4a). CHO-K1 cells were treated with saporin-encapsulated EVs (EVs (25 μg) and SAP (50 μg) in the electroporation condition as described in the Methods section) with or without oligoarginine peptide modifications for 48 h at 37 °C prior to microscope observations and a WST-1 assay (Fig. 4b and c). The modification of EV membranes with R16-EMCS significantly enhanced the cytotoxicity of the saporin encapsulated in EVs (Fig. 4b and c). Although the R8-EMCS-modified EVs showed the highest cellular uptake efficiency of the oligoarginine peptides, the modification of saporin encapsulated in EVs with R8-EMCS showed lower anti-cancer bioactivity than those modified by R16-EMCS (Fig. 4b and c). Supplementary Fig. 10 shows the results of biological activity of R16-EMCS (10 μM) modified EVs (20 μg/ml) with different amounts of loaded saporin (EVs (25 μg) and SAP (0, 5, or 50 μg) in the electroporation condition as described in the Methods section); only saporin (50 μg in the electroporation condition)-loaded EVs decorated with R16-EMCS induced significant cytotoxicity. This result suggests that the enhanced cellular uptake and cytosolic release of saporin originally encapsulated in EVs was attained by the modification of EV membranes with R16-EMCS, and each oligoarginine peptide presented a different efficiency of cytosolic release inside the cells, possibly due to the higher endosomal membrane perturbation ability of R16 than shorter oligoarginines.

## Discussion

In this study, we successfully developed enhanced cellular EV uptake methods using arginine-rich CPPs via the active induction of macropinocytosis. Basic mechanisms of cellular EV uptake have been reported, especially in the endocytotic pathway^26–29^, and membrane proteins of EVs, such as milk fat globule (MFG)-E8/lactadherin, CD11a, CD54, CD9 and CD81 have been shown to possibly participate in the cellular uptake of EVs as ligand proteins^26^. The binding of EVs to the surface of a recipient cell have been also shown to involve the interaction of EV membrane molecules and cellular receptors, including intracellular adhesion molecule 1 (ICAM1), lymphocyte function-associated antigen 1 (LFA1), phosphatidylserine binding to T cell immunoglobulin domain and mucin domain protein 1 (TIM1) or TIM4^27^. The cellular uptake of EVs has been also shown to be dependent on extracellular signal-regulated kinase-1/2 (ERK1/2) and heat shock protein 27 (HSP27) signalling, and ERK1/2 phosphorylation was negatively influenced by caveolin-1 during the internalization of EVs^29^. Therefore, the expression levels of receptors on the recipient cell surface and the ligand proteins on the EV membrane possibly decide the efficacies of cell membrane accumulation of EVs. Ligand-receptor interaction leads to EVs’ binding and accumulation on the recipient cell surface; additionally, receptor-mediated endocytosis induced by receptor activation is important for EVs’ cellular uptake. However, endocytosis, including clathrin-mediated endocytosis and caveolin-mediated endocytosis, has a size limitation for their cellular uptake of approximately 100 nm because the endocytosis are regulated by membrane curvature and the self-assembly of protein scaffolds, including clathrin coats^10, 30^.

However, we recently reported on the active cellular uptake of EVs through the macropinocytosis pathway induced by the stimulation of macropinocytosis-related receptors (e.g., epidermal growth factor receptor) and oncogenic Ras protein^7^. Macropinocytosis can uptake a large volume of extracellular fluid molecules into cells (more than 1 μm in size)^30^. EVs (exosomes) have been shown to be approximately 30–200 nm in size^7, 8, 11, 31–33^, resulting in their low cellular uptake of EVs via endocytosis. EV membranes are also negatively charged^7, 8, 11, 31–33^, leading to their hindered accumulation on negatively charged cell membranes^7, 8, 11^. Very low efficacy of cellular uptake of FITC-dextran-encapsulated EVs without any modification with peptides was also observed (Fig. 2b). Considering these EV characteristics, macropinocytosis is an effective pathway for efficient cellular uptake. Therefore, methodological development for the active induction of macropinocytosis is considered important for intracellular delivery based on EVs.

Arginine-rich CPPs, such as human immunodeficiency virus type 1 (HIV-1) TAT (48–60) peptide and oligoarginine peptides, have been reported to be promising carriers for the intracellular delivery of various bioactive molecules, such as proteins, peptides, and nucleic acids^12, 13^. Macropinocytosis has also been shown to be a crucial pathway for the physiological cellular uptake of arginine-rich CPPs^14–18^. Our research group found that the octaarginine peptide, which is a representative arginine-rich CPPs, induces the clustering of the syndecan-4 proteoglycan on plasma membranes, leading to PKCα binding to the V domain of proteoglycans in the cytosol and the activation of PKCα^19^. The induction of proteoglycan clustering and PKCα activation results in the induction of macropinocytosis and effective cellular uptake of the octaarginine peptide^19^. Proteoglycan dependent cellular uptake of arginine-rich CPPs was also shown^17, 18, 34–36^. From the point of artificial induction of macropinocytosis for the development of EV-based intracellular delivery systems, we have demonstrated here that modification of oligoarginine peptides on EV membranes results in the effective induction of macropinocytosis and cellular EV uptake in this study (Fig. 1).

We propose here a simple experimental technique using oligoarginine peptides with an EMCS-linker for enhancing cellular EV uptake (Fig. 1). The modification of oligoarginine peptides highly enhances cellular EV uptake via the active induction of macropinocytosis without cytotoxicity. The number of arginine residues in the sequence of oligoarginine peptides has been shown to affect macropinocytosis induction, cellular uptake efficiency and membrane penetration^15–20^. In this study, we found that cellular EV uptake was enhanced when the membrane was modified with oligoarginine peptides. Moreover, the number of arginine residues in the modified peptides affected the cellular uptake and cytosolic release of EV content. Interestingly, R8-EMCS modified EVs showed higher cellular-uptake efficacy than that of R16-EMCS modified EVs (Fig. 2). However, in the experiments of cytosolic delivery of a ribosome-inactivating protein, saporin, using oligoarginine peptide-modified EVs, the cytosolic release efficacy of R16-EMCS-modified EVs was much higher than that of R8-EMCS-modified EVs (Fig. 4). This result suggests that each oligoarginine peptide presented a different efficiency of cytosolic release inside the cells, possibly due to the higher endosomal membrane perturbation ability of R16 than that of shorter oligoarginines. We previously reported that the number of arginine residues in the sequence of oligoarginine peptides has been shown to influence their cellular uptake and cytosolic release efficiency, and dodeca- or hexadeca-arginine peptides showed higher cellular uptake and cytosolic release efficacy in comparison to that of octaarginine peptides^20^. Meloni *et al*. also reported that increasing poly-arginine length increased their biological activity^37^. However, in the case of oligoarginine peptide-modified EVs, the gathering of oligoarginine peptides on EVs as scaffolds affects their functionality of cellular uptake and cytosolic release efficacy. Although further studies are needed to elucidate the scaffold effects on the biological functions of arginine-rich CPPs, our methodology using arginine-rich CPPs will strongly contribute to the further development of EV-based intracellular delivery via active macropinocytosis induction to achieve effective and rational cellular EV uptake, considering the characteristics of endocytosis pathways and EVs.

In conclusion, our research results provide fundamental knowledge for the further establishment of EV-based intracellular delivery systems and offer insights into the functionality and applicability of arginine-rich CPPs at the chemical biological boundary.

## Methods

### Peptide synthesis

Chemical synthesis of all peptides was conducted via 9-fluorenylmethyloxycarbonyl (Fmoc) solid-phase peptide synthesis on a Rink amide resin with coupling reagents of 1-hydroxybenzotriazole (HOBt)/2-(1H-Benzotriazole-1-yl)-1,1,3,3-tetramethyluronium hexafluorophosphate (HBTU) (Peptide Institute, Osaka, Japan)/N-methylmorpholine (NMM) as previously described^17, 38^. The Rink amide resin and the Fmoc-amino acid derivatives were purchased from Shimadzu Biotech (Kyoto, Japan) and the Peptide Institute (Osaka, Japan), respectively. To prepare the acetylated peptide, the N-terminus of the peptide resin was acetylated using acetic anhydride in the presence of NMM in dimethylformamide (DMF), as previously reported^39^. Deprotection of the protected peptide and cleavage from the resin was conducted via treatment with a trifluoroacetic acid (TFA)/ethanedithiol (EDT) mixture (95:5) for 3 h at 25 °C, followed by reverse-phase high-performance liquid chromatographic (HPLC) purification. The purity of each peptide was estimated to be >97% on the basis of the analytical HPLC. The structures of the synthesized peptides were confirmed using matrix-assisted laser desorption ionization time-of-flight mass spectrometry (MALDI-TOFMS) (Microflex, Bruker, Billerica, MA, USA).

Ac-CG-R4 (CH_3_-CO-NH-Cys-Gly-(Arg)_4_-amide): MALDI-TOFMS: 843.6 [calcd. for (M + H)^+^: 844.5]. Retention time in HPLC, 7.8 min (column: Cosmosil 5C18-AR-II (4.6 × 150 mm); gradient: 5–50% B in A (A = H_2_O containing 0.1% CF_3_COOH, B = CH_3_CN containing 0.1% CF_3_COOH) over 30 min; flow: 1 mL/min; detection: 220 nm). Yield from the starting resin, 5.1%.

Ac-CG-R8 (CH_3_-CO-NH-Cys-Gly-(Arg)_8_-amide): MALDI-TOFMS: 1468.9 [calcd. for (M + H)^+^: 1468.9]. Retention time in HPLC, 10.6 min (column: Cosmosil 5C18-AR-II (4.6 × 150 mm); gradient: 5–50% B in A (A = H_2_O containing 0.1% CF_3_COOH, B = CH_3_CN containing 0.1% CF_3_COOH) over 30 min; flow: 1 mL/min; detection: 220 nm). Yield from the starting resin, 16.7%.

Ac-CG-R12 (CH_3_-CO-NH-Cys-Gly-(Arg)_12_-amide): MALDI-TOFMS: 2092.8 [calcd. for (M + H)^+^: 2093.3]. Retention time in HPLC, 13.1 min (column: Cosmosil 5C18-AR-II (4.6 × 150 mm); gradient: 5–50% B in A (A = H_2_O containing 0.1% CF_3_COOH, B = CH_3_CN containing 0.1% CF_3_COOH) over 30 min; flow: 1 mL/min; detection: 220 nm). Yield from the starting resin, 5.5%.

Ac-CG-R16 (CH_3_-CO-NH-Cys-Gly-(Arg)_16_-amide): MALDI-TOFMS: 2717.0 [calcd. for (M + H)^+^: 2717.7]. Retention time in HPLC, 14.6 min (column: Cosmosil 5C18-AR-II (4.6 × 150 mm); gradient: 5–50% B in A (A = H_2_O containing 0.1% CF_3_COOH, B = CH_3_CN containing 0.1% CF_3_COOH) over 30 min; flow: 1 mL/min; detection: 220 nm). Yield from the starting resin, 4.4%.

R8 (NH_2_-(Arg)_8_-amide): MALDI-TOFMS: 1266.8 [calcd. for (M + H)^+^: 1266.8]. Retention time in HPLC, 7.1 min (column: Cosmosil 5C18-AR-II (4.6 × 150 mm); gradient: 5–95% B in A (A = H_2_O containing 0.1% CF_3_COOH, B = CH_3_CN containing 0.1% CF_3_COOH) over 30 min; flow: 1 mL/min; detection: 220 nm). Yield from the starting resin, 3.3%.

R16 (NH_2_-(Arg)_16_-amide): MALDI-TOFMS: 2515.0 [calcd. for (M + H)^+^: 2515.7]. Retention time in HPLC, 8.3 min (column: Cosmosil 5C18-AR-II (4.6 × 150 mm); gradient: 5–95% B in A (A = H_2_O containing 0.1% CF_3_COOH, B = CH_3_CN containing 0.1% CF_3_COOH) over 30 min; flow: 1 mL/min; detection: 220 nm). Yield from the starting resin, 9.2%.

### Conjugation of peptides with N-ε-maleimidocaproyl-oxysulfosuccinimide ester (EMCS) linker

For the preparation of EMCS linker-conjugated peptides, each purified Ac-CG-Rn (n = 4, 8, 12, 16) peptide was reacted with EMCS (1.1 equivalents) (Thermo Fisher Scientific Inc., Rockford, IL, USA) in dimethyl formamide for 2 h at room temperature followed by HPLC purification.

EMCS-R4 (CH_3_-CO-NH-Cys(EMCS)-Gly-(Arg)_4_-amide): MALDI-TOFMS: 1232.5 [calcd. for (M + H)^+^: 1231.8]. Retention time in HPLC, 11.6 min (column: Cosmosil 5C18-AR-II (4.6 × 150 mm); gradient: 5–95% B in A (A = H_2_O containing 0.1% CF_3_COOH, B = CH_3_CN containing 0.1% CF_3_COOH) over 30 min; flow: 1 mL/min; detection: 220 nm). Yield from the starting resin, 1.9%.

EMCS-R8 (CH_3_-CO-NH-Cys(EMCS)-Gly-(Arg)_8_-amide): MALDI-TOFMS: 1856.6 [calcd. for (M + H)^+^: 1856.3]. Retention time in HPLC, 10.4 min (column: Cosmosil 5C18-AR-II (4.6 × 150 mm); gradient: 5–95% B in A (A = H_2_O containing 0.1% CF_3_COOH, B = CH_3_CN containing 0.1% CF_3_COOH) over 30 min; flow: 1 mL/min; detection: 220 nm). Yield from the starting resin, 11.2%.

EMCS-R12 (CH_3_-CO-NH-Cys(EMCS)-Gly-(Arg)_12_-amide): MALDI-TOFMS: 2480.7 [calcd. for (M + H)^+^: 2480.7]. Retention time in HPLC, 12.4 min (column: Cosmosil 5C18-AR-II (4.6 × 150 mm); gradient: 5–95% B in A (A = H_2_O containing 0.1% CF_3_COOH, B = CH_3_CN containing 0.1% CF_3_COOH) over 30 min; flow: 1 mL/min; detection: 220 nm). Yield from the starting resin, 2.6%.

EMCS-R16 (CH_3_-CO-NH-Cys(EMCS)-Gly-(Arg)_16_-amide): MALDI-TOFMS: 3105.1 [calcd. for (M + H)^+^: 3105.1]. Retention time in HPLC, 12.4 min (column: Cosmosil 5C18-AR-II (4.6 × 150 mm); gradient: 5–95% B in A (A = H_2_O containing 0.1% CF_3_COOH, B = CH_3_CN containing 0.1% CF_3_COOH) over 30 min; flow: 1 mL/min; detection: 220 nm). Yield from the starting resin, 1.8%.

### Fluorescently labelled peptides

For the preparation of fluorescently labelled peptides, a peptide resin with γ-aminobutyric acid (GABA) at its N-terminus was prepared, and the N-terminus was modified with fluorescein-5-isothiocyanete (FITC) in the presence of N,N-diisopropylethylamine (DIEA) in DMF^40^. Deprotection of the protected peptide, cleavage from the resin, and HPLC purification were conducted as mentioned above.

FITC-EMCS-R4 (FITC-NH-GABA-Cys(EMCS)-Gly-(Arg)_4_-amide): MALDI-TOFMS: 1665.1 [calcd. for (M + H)^+^: 1664.3]. Retention time in HPLC, 13.1 min (column: Cosmosil 5C18-AR-II (4.6 × 150 mm); gradient: 5–95% B in A (A = H_2_O containing 0.1% CF_3_COOH, B = CH_3_CN containing 0.1% CF_3_COOH) over 30 min; flow: 1 mL/min; detection: 220 nm). Yield from the starting resin, 7.2%.

FITC-EMCS-R8 (FITC-NH-GABA-Cys(EMCS)-Gly-(Arg)_8_-amide): MALDI-TOFMS: 2289.4 [calcd. for (M + H)^+^: 2288.7]. Retention time in HPLC, 12.4 min (column: Cosmosil 5C18-AR-II (4.6 × 150 mm); gradient: 5–95% B in A (A = H_2_O containing 0.1% CF_3_COOH, B = CH_3_CN containing 0.1% CF_3_COOH) over 30 min; flow: 1 mL/min; detection: 220 nm). Yield from the starting resin, 2.8%.

FITC-EMCS-R12 (FITC-NH-GABA-Cys(EMCS)-Gly-(Arg)_12_-amide): MALDI-TOFMS: 2912.2 [calcd. for (M + H)^+^: 2913.1]. Retention time in HPLC, 12.3 min (column: Cosmosil 5C18-AR-II (4.6 × 150 mm); gradient: 5–95% B in A (A = H_2_O containing 0.1% CF_3_COOH, B = CH_3_CN containing 0.1% CF_3_COOH) over 30 min; flow: 1 mL/min; detection: 220 nm). Yield from the starting resin, 6.2%

FITC-EMCS-R16 (FITC-NH-GABA-Cys(EMCS)-Gly-(Arg)_16_-amide): MALDI-TOFMS: 3537.7 [calcd. for (M + H)^+^: 3537.5]. Retention time in HPLC, 11.9 min (column: Cosmosil 5C18-AR-II (4.6 × 150 mm); gradient: 5–95% B in A (A = H_2_O containing 0.1% CF_3_COOH, B = CH_3_CN containing 0.1% CF_3_COOH) over 30 min; flow: 1 mL/min; detection: 220 nm). Yield from the starting resin, 3.5%.

FITC-R8 (FITC-NH-GABA-(Arg)_8_-amide): MALDI-TOFMS: 1741.4 [calcd. for (M + H)^+^: 1741.3]. Retention time in HPLC, 11.0 min (column: Cosmosil 5C18-AR-II (4.6 × 150 mm); gradient: 5–95% B in A (A = H_2_O containing 0.1% CF_3_COOH, B = CH_3_CN containing 0.1% CF_3_COOH) over 30 min; flow: 1 mL/min; detection: 220 nm). Yield from the starting resin, 3.6%.

FITC-R16 (FITC-NH-GABA-(Arg)_16_-amide): MALDI-TOFMS: 2990.0 [calcd. for (M + H)^+^: 2990.1]. Retention time in HPLC, 10.8 min (column: Cosmosil 5C18-AR-II (4.6 × 150 mm); gradient: 5–95% B in A (A = H_2_O containing 0.1% CF_3_COOH, B = CH_3_CN containing 0.1% CF_3_COOH) over 30 min; flow: 1 mL/min; detection: 220 nm). Yield from the starting resin, 4.7%.

### Cell cultures

HeLa (human cervical cancer-derived) cells, Chinese hamster ovary (CHO)-K1 cells, and CHO-A745 cells (lacking xylosyltransferase), were purchased from the Riken BRC Cell Bank (Ibaraki, Japan) (HeLa cells) and American Type Culture Collection (Manassas, VA, USA) (CHO-K1 and CHO-A745 cells), respectively. Each cell was cultured in α-MEM (Gibco, Life Technologies Corporation, Grand Island, NY, USA) (HeLa cells) and F-12 nutrient mixture (Ham’s F-12) (CHO-K1 and CHO-A745 cells) (Gibco, Life Technologies Corporation) containing 10% heat-inactivated FBS (Gibco, Life Technologies Corporation). Each cell was grown on 100-mm dishes and incubated at 37 °C under 5% CO_2_.

### HeLa cells stably expressing green fluorescent protein (GFP)-fused CD63

CD63 is an EV (exosomal) membrane marker tetraspanin protein, and we prepared HeLa cells stably expressing GFP-fused CD63 to secrete CD63-GFP-containing EVs (CD63-GFP EVs) as previously reported^7, 8^. The HeLa cells (1 × 10^5^ cells) were plated on a 24-well microplate (Iwaki, Tokyo, Japan) and incubated for 1 day, and they were then transfected with CD63-GFP plasmid (pCT-CD63-GFP, pCMV, Cyto-Tracer, System Biosciences, Mountain View, CA) (800 ng) complexed with Lipofectamine LTX reagent (2 μl) and PLUS reagent (1 μl) (Invitrogen, Life Technologies Corporation, Eugene, OR, USA) in α-MEM containing 10% FBS (200 μl). The cells were also treated with puromycin (3 μg/ml) (LKT Laboratories, St. Paul, MN) for the antibiotic selection of HeLa cells stably expressing CD63-GFP (CD63-GFP-HeLa).

### Isolation of EVs

CD63-GFP-HeLa cells (2 × 10^6^ cells) were seeded onto 100-mm dishes in α-MEM containing 10% EV-free FBS (EXO-FBS, ATLAS biological, Fort Collins, CO, USA) for 3 days. The collection of cell culture medium and isolation of the secreted EVs using ultracentrifugation were conducted as previously reported^21^. The collected cell culture medium was centrifuged (300 × *g*) for 10 min at 4 °C. The supernatant was centrifuged (2,000 × *g*) for 10 min at 4 °C and centrifuged again (10,000 × *g*) for 30 min at 4 °C to remove cell debris. The supernatant was then centrifuged (100,000 × *g*) for 70 min at 4 °C (Himac CP65β, Hitachi, Tokyo, Japan) in duplicate, and the pellet was collected in PBS. The concentrations of isolated EVs were described in terms of their protein concentrations, which were determined using a Pierce BCA protein assay kit (Thermo Fisher Scientific Inc., Rockford, IL, USA).

### Western blotting analysis

To detect EV (exosome) marker proteins, isolated EVs were added to lysis buffer (50 mM Tris-HCl (pH = 7.5), 150 mM NaCl, 0.1% SDS, 1% Triton X-100, and 1% sodium deoxycholate). The boiled samples were separated via 10% SDS-PAGE, transferred onto polyvinylidene fluoride (PVDF) membranes (GE Healthcare, Pittsburgh, PA, USA), and treated with anti-CD9 (EPR2949, Abcam, Cambridge, UK) or anti-CD63 antibody (TS63, Abcam). A secondary antibody labelled with horseradish peroxidase (anti-rabbit IgG HRP-linked whole antibody donkey (GE Healthcare) (for anti-CD9) or anti-mouse IgG HRP NA931V (GE Healthcare) (for anti-CD63)) was used (1:1000 dilution of each antibody in TBS). Immunoreactive species were detected using the Enhanced Chemiluminescence (ECL) Plus Western Blotting Detection System (GE Healthcare) with the Amersham Imager 600 (GE Healthcare).

### Modification of EVs with EMCS-Rn peptides

Synthesized EMCS-Rn (n = 4, 8, 12, 16) peptides (n = 4, 8, 12: final 3.6 mM, n = 16: final 1.8 mM) diluted with H_2_O was added to a solution of EVs (36 μg) in PBS (total 57 μl) and incubated for 30 min at 25 °C. The attachment of FITC-EMCS-Rn (n = 4, 8, 12, 16) to EVs was confirmed using a spectrofluorometer (FP-6200, JASCO, Tokyo, Japan) after the removal of unattached peptides, which was accomplished by washing with PBS and filtration using Amicon Ultra-centrifugal filters (100 K device, Merck Millipore, Billerica, MA, USA).

### Confocal microscopy

CHO-K1 cells (2 × 10^5^ cells, 2 mL) were plated onto a 35-mm glass dish (Iwaki, Tokyo, Japan) and incubated in Ham’s F-12 containing 10% FBS for 24 h at 37 °C under 5% CO_2_. After complete adhesion, the cells were washed with cell culture medium containing 10% FBS and treated with each EV sample (100 μl/well). The cells were stained with Hoechst 33342 dye (Invitrogen; 5 μg/ml) for 15 min at 37 °C before cell washing. The cells were then washed with fresh cell culture medium and analysed using a FV1200 confocal laser scanning microscope (Olympus, Tokyo, Japan) equipped with a 40x objective without cell fixation.

To visualize the lamellipodia formation of cellular actin, the cells were fixed with 4% paraformaldehyde at room temperature for 30 min and washed with PBS after the cells were treated with each EV sample (100 μl/well). The cells were then treated with 0.1% Triton X-100 (100 μl/well in PBS) at room temperature for 5 min and again washed with PBS. Cellular F-actin was stained with rhodamine-phalloidin (2.5 μl (300 units) in PBS (97.5 μl)/well) (Molecular Probes) for 20 min at 4 °C, and the cells were washed with PBS before analysis using a FV1200 confocal laser scanning microscope (Olympus) equipped with a 40× objective.

### Flow cytometer

CHO-K1 cells (1.4 × 10^5^ cells, 1 mL) were plated onto a 24-well microplate (Iwaki) and incubated in Ham’s F-12 containing 10% FBS for 24 h at 37 °C under 5% CO_2_. After complete adhesion, the cells were washed with cell culture medium containing 10% FBS and treated with each EV sample (600 μl/well) before washing with 0.5 mg/ml heparin in PBS (triple washing, 200 μl). The cells were then treated with 0.01% trypsin at 37 °C for 10 min before the addition of PBS (200 μl) and then centrifuged at 3,000 rpm (800 × *g*) for 5 min at 4 °C. After removal of the supernatant, the cells were washed with PBS (400 μl) and centrifuged at 3,000 rpm for 5 min at 4 °C. This washing cycle was repeated, and the cells were suspended in PBS (400 μl) and subjected to fluorescence analysis with a guava easyCyte (Merck Millipore) flow cytometer using 488-nm laser excitation and a 525-nm emission filter. Live cells (10,000 cells/sample) for the detection of cellular fluorescence intensity were quantified based on forward-scattering and side-scattering analyses.

In the assessment of macropinocytosis, the cells were treated with the macropinocytosis marker FITC-dextran (molecular weight: 70,000, 0.25 mg/ml, Sigma-Aldrich) and each exosomal sample (600 μl/well) for 3 h at 37 °C under 5% CO_2_ prior to the flow cytometry analysis.

In the macropinocytosis inhibition assay, the cells were pretreated with 5-(N-ethyl-N-isopropyl) amirolide (100 μM, 600 μl/well; EIPA, Sigma-Aldrich) in Ham’s F-12 containing 10% FBS for 30 min at 37 °C prior to the treatment with each sample (600 μl/well) in Ham’s F-12 containing 10% FBS in the presence or absence of EIPA (100 μM) for 1 h at 37 °C.

### Electron microscopy

EV samples suspended in PBS (30 μg/ml) were dropped onto a carbon-coated grid (400 mesh) and washed with distilled water. Uranyl acetate was applied to the grid and left for 10 s at room temperature. Next, the reagent was removed with filter paper and dried prior to imaging with a transmission electron microscope (TEM) (JEM1200EX, JEOL, Tokyo, Japan).

### Zeta-potential and particle size

The zeta-potential and particle size of the EV samples diluted in PBS (58.2 μg/ml) were determined using a zeta-potential and particle size analyser ELSZ-DN2 (Otsuka Electronics, Osaka, Japan) according to the manufacturer’s instructions.

### Particle numbers of EVs

The number of isolated EVs was counted using a qNano nanoparticle/microparticle analyzer (iZON Science, Oxford, United Kingdom) according to the manufacturer’s instructions.

### Preparation of FITC-labelled saporin

To prepare the FITC-labelled saporin, saporin (200 μg, saporin from *Saponaria officinalis* seeds, Sigma-Aldrich) dissolved in H_2_O (100 μl) was reacted with 2 equivalents of FITC (Sigma-Aldrich) dissolved in dimethyl sulfoxide (10 μl) and N,N-diisopropylethylamine (0.5 μl) at 30 °C for 2 h as previously reported^7, 8^. To remove the unreacted FITC, gel filtration on a Sephadex G-25 column (PD-10, GE Healthcare) was performed before lyophilization. The protein concentration was determined using a Pierce BCA protein assay kit.

### Encapsulation of fluorescently labelled dextran and saporin into EVs

To load fluorescently labelled dextran into EVs, the EVs (25 μg) were mixed with FITC-labelled dextran (molecular weight: 70,000) (Sigma-Aldrich) or saporin (0, 5, 50 μg each) in PBS (100 μl). After electroporation (poring pulse: twice pulse (100 V, 5 msec); transfer pulse: five pulse (20 V, 50 msec) in a 1-cm electroporation cuvette at room temperature using a super electroporator NEPA21 Type II (NEPA Genes, Tokyo, Japan), removal of unencapsulated FITC-dextran or saporin was accomplished by washing and filtration using Amicon Ultra centrifugal filters (100 K device) as previously reported^7, 8^. Loading of FITC-labelled dextran or FITC-labelled saporin into EVs was confirmed using a spectrofluorometer (FP-6200, JASCO, Tokyo, Japan). The electroporation method resulted in the encapsulation of FITC-dextran (376.7 ng/ml) in 20 μg/ml of EVs. The efficiency of dextran encapsulation into EVs was calculated at 0.9%. The concentration of saporin encapsulated in 20 μg/ml EVs was estimated at approximately 65 ng/ml using the FITC-labelled saporin. The efficiency of saporin encapsulation into EVs was calculated at 0.1%.

### Cell viability (WST-1 assay)

Analysis of cell viability was conducted using the WST-1 (4-[3-(4-iodophenyl)-2-(4-nitrophenyl)-2H-5-tetrazolio]-1,3-benzene disulfonate) assay as previously described^7, 8^. CHO-K1 cells (1 × 10^4^ cells, 100 μl) were incubated in 96-well microplates in Ham’s F-12 containing 10% FBS for 24 h at 37 °C under 5% CO_2_. The cells were then treated with each EV sample (50 μl) at 37 °C under 5% CO_2_. After the sample treatment, WST-1 reagents (10 μl) were added to each well and the samples were incubated for 40 min at 37 °C. The absorbencies at 450 nm (A450) and 620 nm (A620) were measured, and the value obtained by subtracting A620 from A450 corresponded to the viable cell number.

### Statistical analyses

All statistical analyses were performed using GraphPad Prism software (ver. 5.00; GraphPad, San Diego, CA, USA). For comparisons of two groups, unpaired Student’s t-test was used for verification of the equal variances via an F-test. Welch’s correction was performed when the variances across groups were assumed to be unequal. For multiple comparison analyses, a one-way analysis of variance (ANOVA) followed by Dunnett’s or Tukey’s multiple comparison test was used. Differences were considered significant when the calculated p-value was <0.05.

## Electronic supplementary material

Supplementary information
