# Supplementary material for: Arginine-rich cell-penetrating peptide-modified extracellular vesicles for active macropinocytosis induction and efficient intracellular delivery
Source: Sci Rep. 2017 May 16;7:1991. doi: 10.1038/s41598-017-02014-6 (PMC5434003; doi:10.1038/s41598-017-02014-6)
Supplement: Supplementary file 1 — Supplementary information [file 41598_2017_2014_MOESM1_ESM.pdf]

Scientific Reports

Supplementary information

Title:

Arginine-rich cell-penetrating peptide-modified extracellular vesicles for active macropinocytosis induction and efficient intracellular delivery

Authors:

Ikuhiko Nakase,[a,]* Kosuke Noguchi,[a,b] Ayako Aoki,[a,b] Tomoka Takatani-Nakase,[c] Ikuo Fujii,[b] Shiroh Futaki[d]

Affiliations:

[a]Nanoscience and Nanotechnology Research Center, Research Organization for the 21st Century, Osaka Prefecture University, 1-2, Gakuen-cho, Naka-ku, Sakai, Osaka 599-8570, Japan

[b]Graduate School of Science, Osaka Prefecture University, 1-1, Gakuen-cho, Naka-ku, Osaka 599-8531, Japan

[c]Department of Pharmaceutics, School of Pharmacy and Pharmaceutical Sciences, Mukogawa Women's University, 11-68, Koshien Kyuban-cho, Nishinomiya, Hyogo 663-8179, Japan

[d]Institute for Chemical Research, Kyoto University, Uji, Kyoto 611-0011, Japan

*Please address correspondence and requests for materials to I.N. (E-mail: i-nakase@21c.osakafu-u.ac.jp).

Supplementary Tables

Peptides	MALDI-TOFMS (m/z)	Calcd. for $(M+H)^+$
Ac-CG-R4 (CH_3-CO-NH-Cys-Gly-$(Arg)_4$-amide)	843.6	844.5
Ac-CG-R8 (CH_3-CO-NH-Cys-Gly-$(Arg)_8$-amide)	1468.9	1468.9
Ac-CG-R12 (CH_3-CO-NH-Cys-Gly-$(Arg)_{12}$-amide)	2092.8	2093.3
Ac-CG-R16 (CH_3-CO-NH-Cys-Gly-$(Arg)_{16}$-amide)	2717.0	2717.7
R8 (NH_2-$(Arg)_8$-amide)	1266.8	1266.8
R16 (NH_2-$(Arg)_{16}$-amide)	2515.0	2515.7
EMCS-R4 (CH_3-CO-NH-Cys(EMCS)-Gly-$(Arg)_4$-amide)	1232.5	1231.8
EMCS-R8 (CH_3-CO-NH-Cys(EMCS)-Gly-$(Arg)_8$-amide)	1856.6	1856.3
EMCS-R12 (CH_3-CO-NH-Cys(EMCS)-Gly-$(Arg)_{12}$-amide)	2480.7	2480.7
EMCS-R16 (CH_3-CO-NH-Cys(EMCS)-Gly-$(Arg)_{16}$-amide)	3105.1	3105.1
FITC-EMCS-R4 (FITC-NH-GABA-Cys(EMCS)-Gly-$(Arg)_4$-amide)	1665.1	1664.3
FITC-EMCS-R8 (FITC-NH-GABA-Cys(EMCS)-Gly-$(Arg)_8$-amide)	2289.4	2288.7
FITC-EMCS-R12 (FITC-NH-GABA-Cys(EMCS)-Gly-$(Arg)_{12}$-amide)	2912.2	2913.1
FITC-EMCS-R16 (FITC-NH-GABA-Cys(EMCS)-Gly-$(Arg)_{16}$-amide)	3537.7	3537.5
FITC-R8 (FITC-NH-GABA-$(Arg)_8$-amide)	1741.4	1741.3
FITC-R16 (FITC-NH-GABA-$(Arg)_{16}$-amide)	2990.0	2990.1

***Supplementary Table 1.* MALDI-TOFMS data of synthesized oligoarginine peptides.**

Supplementary Figures

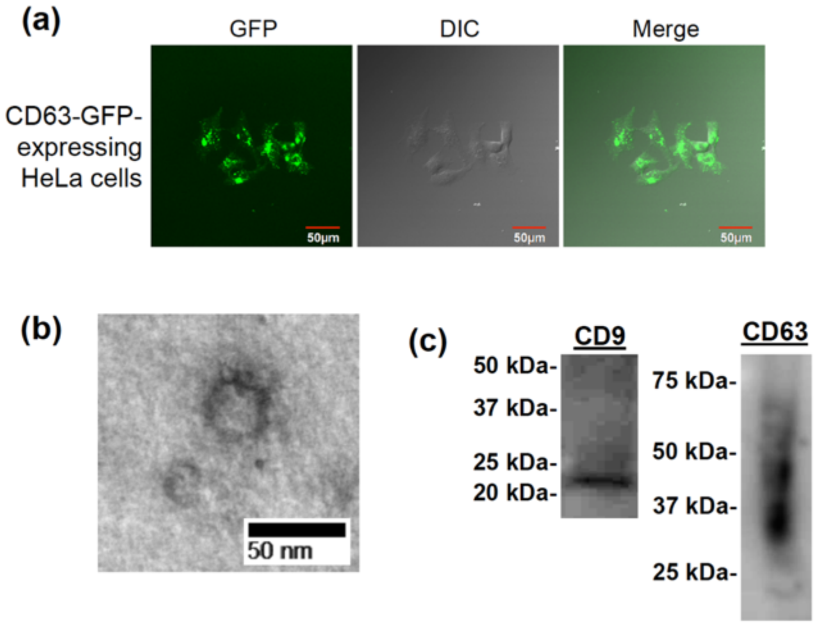

Supplementary Figure 1. **Cellular secretion of CD63-GFP-EVs from HeLa cells stably expressing CD63-GFP.** (**a**) Confocal microscopy observations of HeLa cells stably expressing CD63-GFP. Scale bar: 50 μm. (**b**) TEM observations of isolated CD63-GFP-EVs. Scale bar: 50 nm. (**c**) Western blot analyses showing EVs secreted from HeLa cells. The CD9 and CD63 EV (exosome) marker proteins were detected as described in the Methods section. Immunoreactive species were detected using anti-CD9 and anti-CD63 at approximately 23 kDa and 30 ~ 70 kDa, respectively, via an SDS-PAGE analysis.

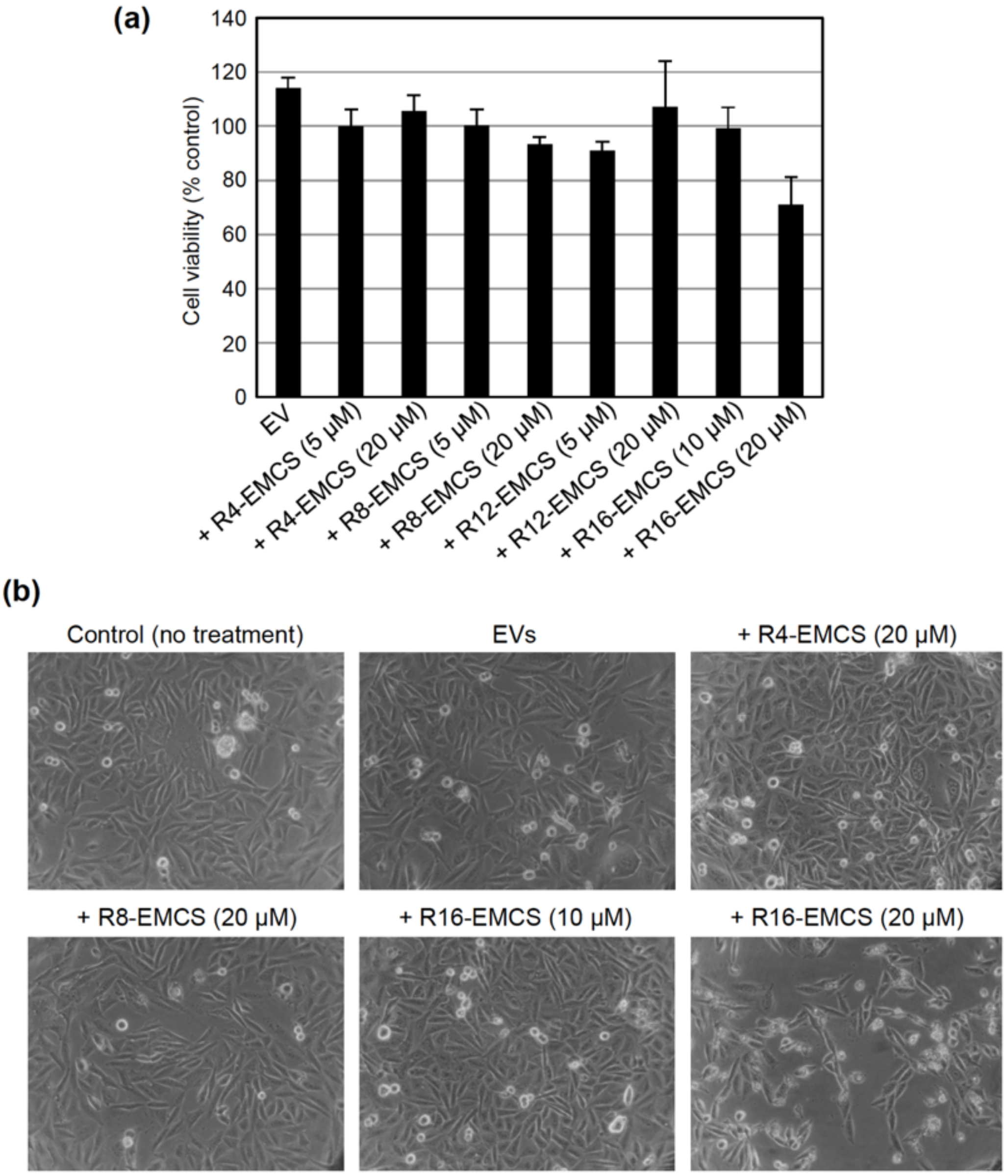

Supplementary Figure 2. **Cell viability.** (**a**) CHO-K1 cells were treated with EVs (20 μg/ml) modified with Rn-EMCS (n = 4, 8, 12, 16: 5 ~ 20 μM) for 24 h at 37°C prior to the WST-1 assay. The data are expressed as the average (± SD) of three experiments. (**b**) Microscope observations of CHO-K1 cells treated with EVs modified with Rn-EMCS under the same experimental conditions as (**a**).

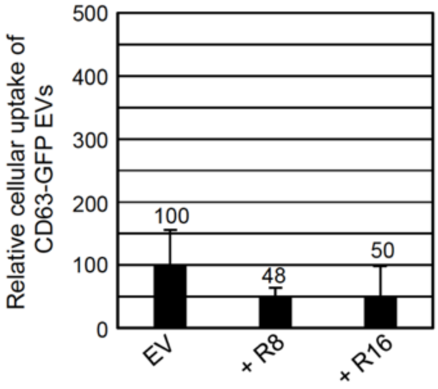

Supplementary Figure 3. **Cellular uptake efficiency of EVs mixed with oligoarginine peptides without EMCS linkers.** Relative cellular uptake of CD63-GFP EVs (20 μg/ml) mixed with Rn (n = 8: 20 μM, n = 16: 10 μM) in CHO-K1 cells for 24 h at 37°C according to the flow cytometry analysis. The data are expressed as the average (± SD) of three experiments.

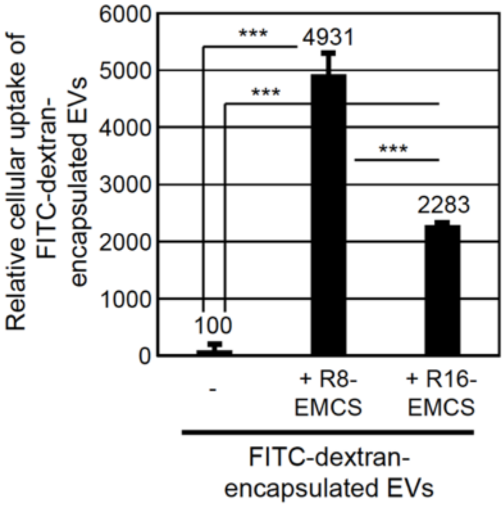

Supplementary Figure 4. **Cellular uptake efficiency of EVs modified with oligoarginine peptides.** Relative cellular uptake of FITC-dextran-encapsulated EVs (20 μg/ml) modified with Rn-EMCS (n = 8: 20 μM, n = 16: 10 μM) in CHO-K1 cells for 24 h at 37°C according to the flow cytometry analysis. The data are expressed as the average (± SD) of three experiments. ***$p < 0.001$.

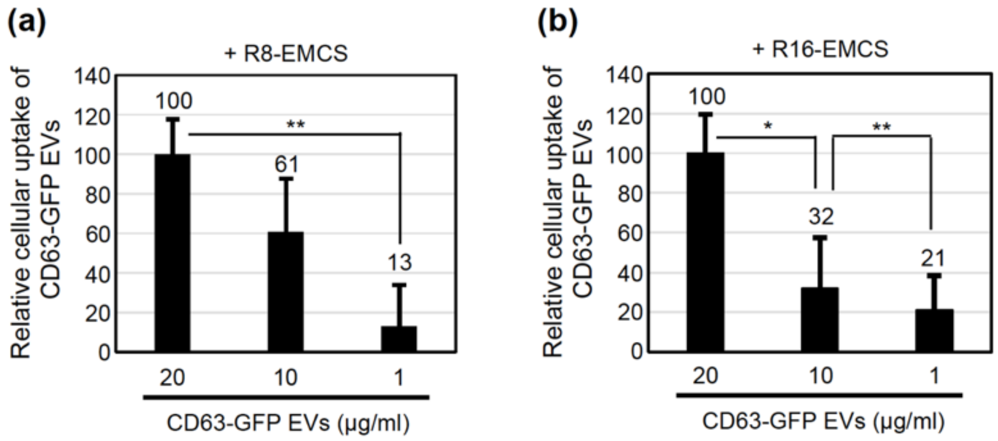

Supplementary Figure 5. **Cellular uptake efficiency of EVs upon modification with oligoarginine peptides.** Relative cellular uptake of CD63-GFP EVs (20, 10 or 1 μg/ml) mixed with Rn (n = 8: 20 μM (**a**), n = 16: 10 μM (**b**)) in CHO-K1 cells for 24 h at 37°C according to flow cytometry analysis. The data are expressed as the average (± SD) of three experiments. *$p < 0.05$, **$p < 0.01$.

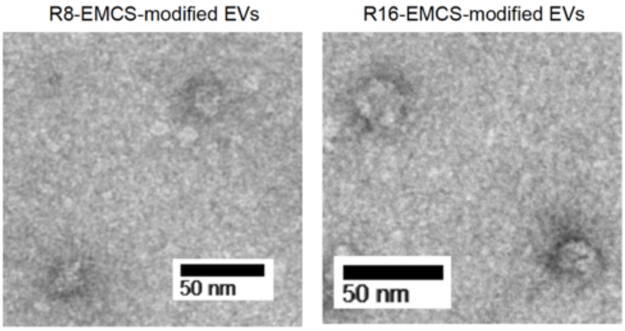

Supplementary Figure 6. **TEM observations of R8- or R16-EMCS-modified CD63-GFP EVs.** Scale bar: 50 nm.

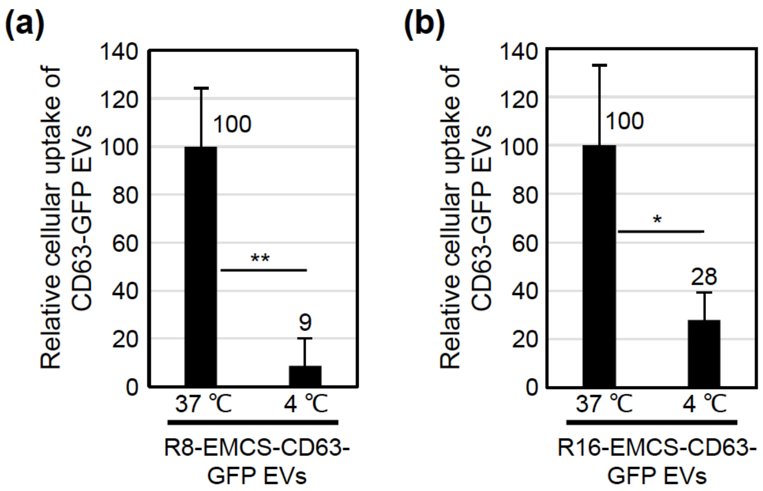

Supplementary Figure 7. **Flow cytometry analysis of the cellular uptake of EVs under the experimental conditions of endocytosis prevention.** (**a**, **b**) Relative cellular uptake of CD63-GFP EVs (20 μg/ml) modified with Rn-EMCS ((**a**) n = 8: 20 μM, (**b**) n = 16: 10 μM) for 2 h at 37°C or 4°C for the prevention of endocytosis, which was analysed via flow cytometry. The data are expressed as the average (± SD) of three experiments. *$p < 0.05$, **$p < 0.01$.

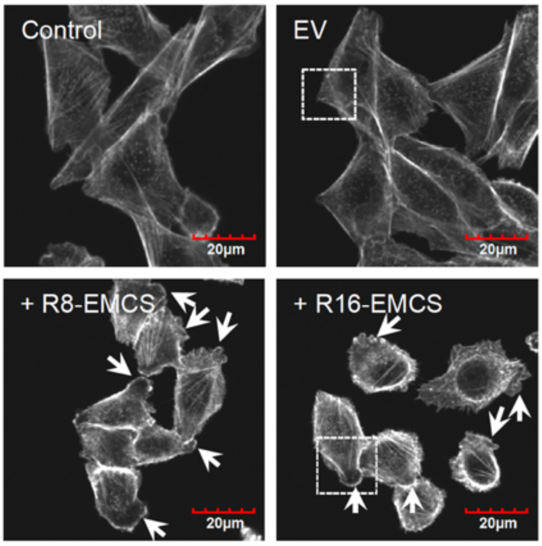

Supplementary Figure 8. **Enhanced lamellipodia formations by the treatment of oligoarginine-modified EVs.** Confocal microscope observation of CHO-K1 cells treated with EVs (20 μg/ml) modified with R8-EMCS (20 μM), R16-EMCS (10 μM), or EVs without the peptide modification for 20 min at 37°C. Cellular staining with rhodamine-phalloidin was conducted to visualize F-actin prior to the observation. The arrows indicate representative lamellipodia formations. Scale bar: 20 μm. Areas of white squares are enlarged in Figure 3f and g.

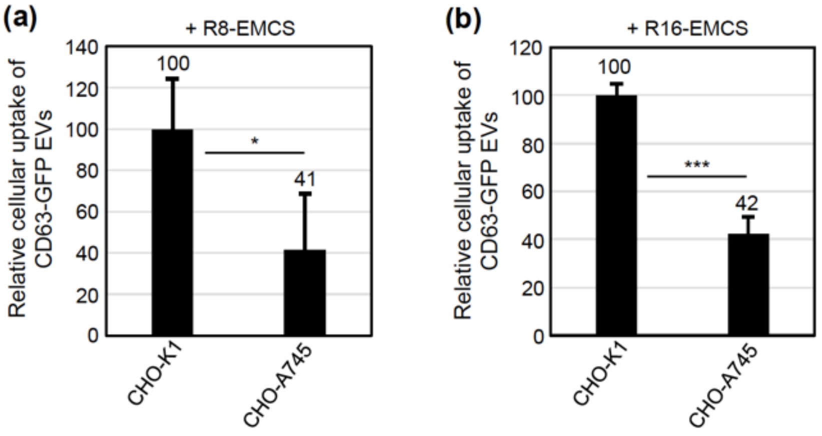

Supplementary Figure 9. **Cellular uptake efficiency of EVs modified with oligoarginine peptides into all glycosaminoglycan-deficient CHO-A745 cells.** Relative cellular uptake of FITC-dextran-encapsulated EVs (20 µg/ml) modified with Rn-EMCS (n = 8: 20 µM (**a**), n = 16: 10 µM (**b**)) in CHO-K1 or all glycosaminoglycan-deficient CHO-A745 cells for 24 h at 37°C according to flow cytometry analysis. The data are expressed as the average (± SD) of three experiments. *$p < 0.05$, ***$p < 0.001$.

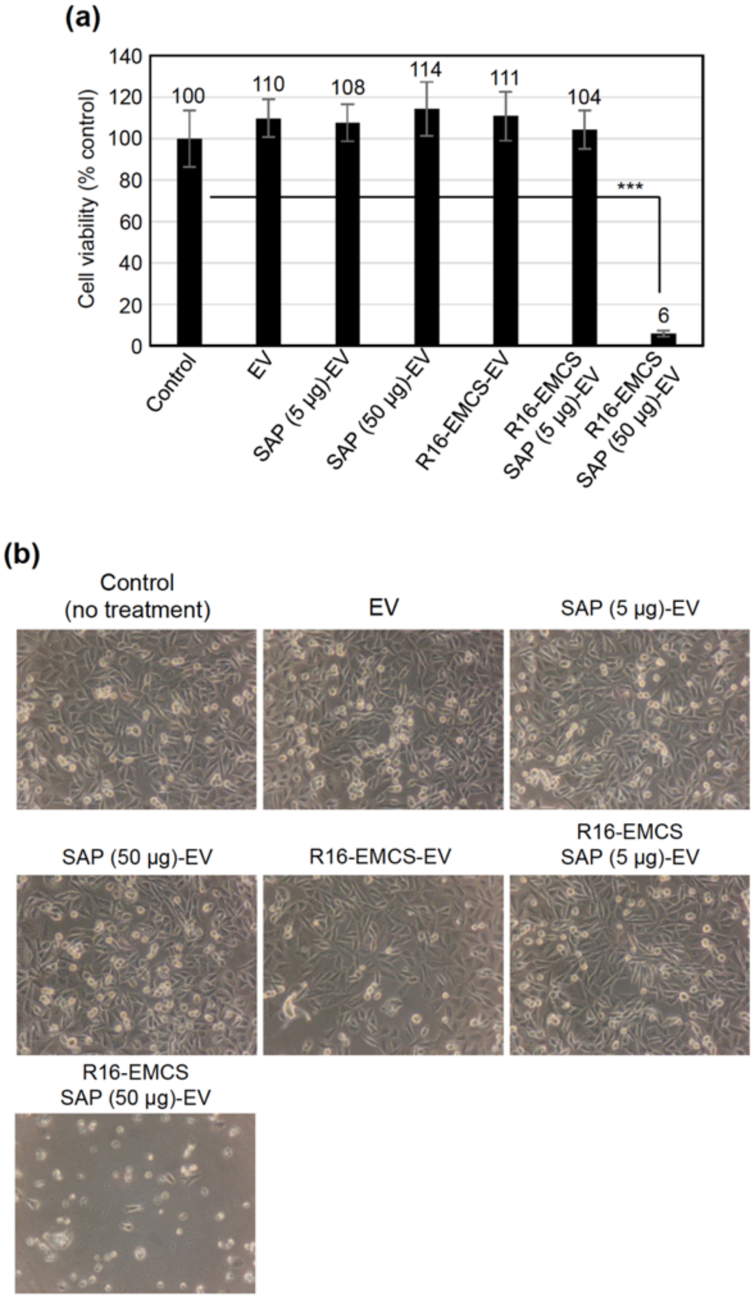

Supplementary Figure 10. **Increased anti-cancer activity of saporin encapsulated in EVs modified by R16-EMCS.** (**a**) CHO-K1 cells were treated with SAP encapsulated in EVs (20 μg/ml) (EVs (25 μg) and SAP (0, 5, or 50 μg) in the electroporation condition as described in the Methods section) with or without modification by R16-EMCS (10 μM) for 48 h at 37°C. Cell viability was then analysed using a WST-1 assay. The data are expressed as the average (± SD) of three experiments. ***p < 0.001. (**b**) Microscope observations of CHO-K1 cells treated with EV samples under the same experimental conditions as (**a**).
